# Mutations in UMOD Contribute to the Pathogenesis of ADTKD‐UMOD by Influencing the Function of Complement Factor H

**DOI:** 10.1111/jcmm.71025

**Published:** 2026-01-14

**Authors:** Qiuyu Xie, Lufeng Bai, Kunjing Gong, Nan Hu, Yuqing Chen

**Affiliations:** ^1^ Renal Division Peking University First Hospital Beijing China; ^2^ Institute of Nephrology Peking University Beijing China; ^3^ Key Laboratory of Renal Disease Ministry of Health of China Beijing China; ^4^ Key Laboratory of CKD Prevention and Treatment Ministry of Education of China Beijing China; ^5^ Research Units of Diagnosis and Treatment of Immune‐Mediated Kidney Diseases Chinese Academy of Medical Sciences Beijing China; ^6^ Joint International Research Center of Translational and Clinical Research Beijing China

**Keywords:** activation, affinity, autosomal dominant tubulointerstitial kidney disease, complement factor H, mutations, uromodulin

## Abstract

Tubular atrophy and interstitial fibrosis are basic renal pathological changes in autosomal dominant tubulointerstitial kidney disease (ADTKD). Reduced secretion or abnormal structure of uromodulin (UMOD) are recognised pathogenic factors of ADTKD. Studies show uromodulin binds complement factor H (cFH), enhancing its ability to inhibit complement activation. Overactivation of the complement system contributes to tubulointerstitial injury. Therefore, exploring the UMOD–tubulointerstitial fibrosis link may aid in the development of treatment for ADTKD‐UMOD. Immunofluorescence staining detected complement deposition in patients' kidneys. Uromodulin's binding affinity for cFH was assessed using microthermophoresis. The effect of this binding on cFH function was analysed using C3b degradation and erythrocyte hemolysis tests. Recombinant wild‐type and mutant uromodulin proteins were expressed and tested using the aforementioned methods. Complement factor B was detected in the kidneys of patients with ADTKD‐UMOD. Patient‐derived uromodulin showed reduced binding to cFH and decreased capacity to assist in C3b cleavage and hemolysis inhibition. Recombinant wild‐type uromodulin significantly enhanced C3b cleavage (*p* < 0.001) and inhibited hemolysis (*p* < 0.01). Uromodulin mutants showed reduced binding to cFH and limited ability to promote C3b degradation, with no significant hemolysis inhibition. Impaired interactions between mutants and cFH may lead to insufficient inhibition of complement activity, triggering tubulointerstitial fibrosis.

## 1 Introduction

Mutations in the *UMOD* gene are recognised as important pathogenic factors in autosomal dominant tubulointerstitial kidney disease (ADTKD) [1]. These mutations lead to delayed peptide maturation and prevent uromodulin (UMOD)'s transport from the endoplasmic reticulum to the Golgi apparatus, thereby hindering extracellular secretion [2]. Tubular atrophy and interstitial fibrosis are the basic pathological changes observed in the kidneys of patients with ADTKD‐UMOD [3]. Reduced extracellular secretion or abnormal structure of uromodulin appears to be a critical factor contributing to the development of renal fibrosis. Some studies indicate that trapped uromodulin can induce endoplasmic reticulum stress, cellular damage and apoptosis [4, 5, 6, 7, 8, 9]. However, other studies have shown that certain UMOD mutations do not result in reduced uromodulin secretion [10], suggesting other pathogenic mechanisms for *UMOD* gene defects.

Evidence suggests that uromodulin can bind to cytokines and complement components, including IL‐1, TNF‐α, IL‐2, IL‐8, C1, C1q and C3 [11, 12, 13, 14, 15], and interact with neutrophils, monocytes, lymphocytes and myeloid dendritic cells [15, 16, 17, 18]. Previous studies have also demonstrated that uromodulin can bind to complement factor H (cFH), enhancing its activity as a cofactor for factor I and inhibiting complement activation by promoting complement degradation [19, 20]. Overactivation of the complement system is involved in tubulointerstitial injury [21]. As a critical regulator of the complement alternative pathway, cFH [22] serves as an important protective factor against renal tubulointerstitial injury by binding to renal tubular epithelial cells and inhibiting complement hyperactivation [23].

Therefore, investigating whether complement activation is a potential link between uromodulin and tubulointerstitial fibrosis may help to identify therapeutic strategies for ADTKD‐UMOD. Given the relationship between uromodulin and cFH, we speculated that *UMOD* mutations might affect cFH function by reducing its interaction with uromodulin, thereby contributing to interstitial fibrosis.

In this study, we observed activation of the alternative complement pathway in a patient with ADTKD, along with a weakened interaction between cFH and uromodulin. We constructed recombinant wild‐type and mutant UMOD proteins based on genetic sequencing of previously identified patients with ADTKD and tested whether these UMOD mutations reduced their binding affinity for cFH and affected cFH function.

## 2 Materials and Methods

### 2.1 Patients With ADTKD‐UMOD

In this study, paraffin and frozen sections, as well as urine samples, were obtained from a patient diagnosed with ADTKD who underwent a renal biopsy. Additionally, we constructed uromodulin mutants with this one and five other UMOD mutations identified in our previously reported patients with ADTKD‐UMOD [24]. The baseline and follow‐up data are summarised in Table 1.

**TABLE 1:** Manifestations of ADTKD‐UMOD patients with different *UMOD* gene mutations [24, 29].

Patient	*UMOD* mutation	Gender	Age at diagnosis	Creatine (μmol/L)	Uric acid (μmol/L)	eGFR mL/min/1.73 m^2^	Family medical history	Follow‐up (year)	ESRD (yes/no)
1	Cys35Tyr	Male	20	163	749	53.1	No	4	No
2	Asn38Ile	Female	38	155	253	36.3	Yes	2	Yes
3	Leu66Pro	Male	44	203	380	24.7	Yes	5	No
4	Pro236Gln	Female	24	230	606	24.9	No	2	Yes
5	Cys287Phe	Female	41	182	474	29.3	Yes	4	No
YHR	Cys94Arg	Female	13	215	441	25.1	No	3	Yes

### 2.2 Immunofluorescence Staining

Frozen sections from a patient with ADTKD‐UMOD were used for immunofluorescence analysis. The sections were blocked with 3% bovine serum albumin (BSA) for 1 h, followed by incubation with primary antibodies, including cFB (Proteintech, China), C3 (Dako, Denmark), C1q (Dako, Denmark) and IgG (Southern Biotech, USA), at 37°C for 30 min. Subsequently, they were incubated with an anti‐rabbit secondary antibody conjugated with Alexa Fluor 488 (Biyuntian, China) at 37°C for 30 min. The sections were washed with PBS and mounted using a medium containing DAPI (Zhongshanjinqiao, China), followed by examination under a Zeiss LSM 780 confocal microscope at 400× magnification (Zeiss, Germany). Semi‐quantitative scoring was performed independently by two pathologists who were blinded to the clinical data. The intensity of specific fluorescence signals was graded on a scale from 0 to 3+: 0 (negative), 1+ (faint), 2+ (distinct) and 3+ (bright). Staining was considered positive if the score was ≥ 1+.

### 2.3 Immunohistochemistry

Paraffin sections were deparaffinised and hydrated using xylene and graded alcohols. Antigen retrieval was performed by high‐pressure heating in EDTA buffer (pH 9.0), followed by cooling to room temperature. The sections were blocked with 3% BSA for 1 h, then incubated overnight with anti‐C4d antibody (BIO‐RAD, USA) at 4°C. Subsequently, they were incubated with an HRP‐conjugated secondary antibody (zhongshanjinqiao, China) at room temperature for 1 h and then reacted with DAB chromogen solution. Counterstaining was carried out with haematoxylin, differentiated with hydrochloric acid‐alcohol and blued in lithium carbonate solution. Finally, the sections were dehydrated through a graded alcohol series, cleared in xylene and mounted with mounting medium for examination under a microscope. Semi‐quantitative scoring was performed independently by two pathologists who were blinded to the clinical data. The distribution area of specific positive signals was graded on a scale from 0 to 3+: 0 (< 5%), 1+ (5%–25%), 2+ (26%–50%) and 3+ (> 50%). Staining was considered positive if the score was ≥ 1+.

### 2.4 Uromodulin Isolation From Healthy Individuals and Patients

Uromodulin was isolated according to the protocol from a previous study [25]. Briefly, urine sample (1 L) was collected, mixed with 20 g of diatomaceous earth (Celite 521, Acros Organics, Belgium) and stirred at 4°C for 20 min. The mixture was then filtered under gentle vacuum aspiration using a filter paper (15 cm, Whatman, England). After filtration, the diatomaceous earth was washed with 1 L of 0.02 M PBS. The diatomaceous earth was subsequently mixed with 150 mL of deionised water at 4°C for 30 min and then centrifuged at 4°C and 20,000 *g* for 30 min. The supernatant was collected while the pellet was discarded. PB buffer and NaCl were added to the supernatant to a final concentration of 0.025 mol/L and 0.14 mol/L respectively. After complete dissolution of NaCl, 5 g of diatomaceous earth was added, and the mixture was stirred at 4°C for 20 min. The suspension was filtered using a filter paper (8 cm, Whatman, England). The diatomaceous earth was then washed with 200 mL of 0.02 M PBS. The filtered diatomaceous earth was resuspended in 50 mL of deionised water and stirred for 30 min at 4°C. The mixture was centrifuged at 4°C and 20,000 *g* for 30 min. The supernatant was dialysed overnight at 4°C and filtered through a 0.2 μm membrane (SLGP033RB, Milipore) in the following day. Finally, the solution was concentrated via 30,000 NMWL filter (AmiconTM Ultra‐15; Milipore), and the protein was lyophilised and stored at −80°C.

### 2.5 Expression and Purification of Recombinant Uromodulin Proteins

The pCAG‐GFP plasmid was used to construct a vector containing the N‐terminal signal peptide sequence (SP) of the *UMOD* gene and a His‐tag (pCAG‐SP‐6xHis‐GFP). The SP sequence was cloned, and a His‐tag was appended to its C‐terminus. Simultaneously, an AsiSI restriction endonuclease site was introduced, and the fragment was amplified via PCR. Subsequently, both the pCAG‐GFP plasmid and the purified PCR product were digested with EcoRI and SmaI restriction enzymes at 37°C for 2 h. Target DNA fragments were separated by agarose gel electrophoresis and recovered using a kit. The DNA fragment and vector were ligated at 16°C using T4 ligase to obtain pCAG‐SP‐6xHis‐GFP.

The *UMOD* gene fragment was amplified using cDNA clones of human uromodulin (from OriGene), corresponding to its transcript variant 1 (NM_003361), as a template. The obtained plasmid and the fragment were digested with AsiSI and NotI restriction enzymes. Following digestion, the GFP sequence in the pCAG‐SP‐6xHis‐GFP vector was removed, and the recombinant DNA fragment (Sequence S1, Supporting Information S1) was inserted to create a plasmid containing the target fragment (Figure S1).

Next, we excised the target fragment and synthesised mutant fragments (Cys35Tyr, Asn38Ile, Leu66Pro, Pro236Gln and Cys287Phe) using the recombinant wild‐type uromodulin fragment (UMOD‐FLR1) as the template, based on the mutations found in patients with ADTKD. The subclonal fragments were ligated into a vector to obtain mutant fragments (Shenggong Biotechnology Co. Ltd. Shanghai, China). Sequences of the fragments inserted into the plasmids were verified by sequencing.

Plasmids containing the target UMOD DNA fragment were transfected into HEK293‐FT cells, which lack the hepsin enzyme that cleaves uromodulin [26], using a transfection reagent (Roche, Switzerland). Cells were cultured in Dulbecco's Modified Eagle Medium supplemented with 10% fetal bovine serum (Gibco, USA). After 48–60 h, the supernatant was collected and centrifuged at 3000 rpm for 10 min, and the precipitate was discarded. The supernatant was then filtered through a 0.2 μm filter membrane, concentrated by centrifugation at 3000 rpm using a 30,000 NMWL filter (Amicon Ultra‐15, Millipore), mixed with binding buffer (Na_2_HPO_4_·12H_2_, 16 mM; NaH_2_PO_4_·2H_2_O, 4 mM; NaCl, 500 mM; imidazole, 5 mM; pH 7.4) and combined with His‐affinity Ni beads (Beaver, Suzhou) at 4°C for 90 min. The magnetic beads were washed twice with washing buffer (Na_2_HPO_4_·12H_2_, 16 mM; NaH_2_PO_4_·2H_2_O, 4 mM; NaCl, 500 mM; imidazole, 50 mM; pH 7.4), and the proteins bound to the magnetic beads were eluted with elution buffer (Na_2_HPO_4_·12H_2_, 16 mM; NaH_2_PO_4_·2H_2_O, 4 mM; NaCl, 500 mM; imidazole, 200 mM; pH 7.4). The eluents were filtered through a 0.2 μm filter membrane and dialysed overnight in deionised water at 4°C. Dialysed protein samples were concentrated by ultrafiltration at 3000 rpm using a 30,000 NMWL filter. Protein concentration was quantified. Sample purity and the presence of uromodulin were evaluated by silver staining and Western blotting, respectively, using an anti‐uromodulin antibody (R&D Systems, USA).

### 2.6 Microscale Thermophoresis (MST)

The binding capacity of recombinant uromodulin proteins to complement factor H (cFH) was assessed using MST (Monolith NT.115, NanoTemper, Germany) as described previously [27]. Recombinant uromodulin proteins were labelled with a red fluorescent dye and maintained at a concentration of 100 nM (Monolith NTTM Protein Labelling Kit). cFH was diluted from an initial concentration of 16.8 μmol/L. The MST power was set to 40%, and the LED excitation power was adjusted to 100%. Binding affinity was measured in standard‐treated capillaries, and the data were analysed using the Hill model of the MO Affinity Analysis software.

### 2.7 Cofactor Activity Analysis of cFH

The cofactor activity of cFH was measured as previously described [19]. Our assay was performed in a final volume of 20 μL, comprising cFH (0.5 μg, Merck, Kenilworth), factor I (50 ng, Merck), C3b (2.5 μg, Merck) and uromodulin (8 μg). The mixture was incubated at 37°C for 40 min. Negative controls were prepared in the absence of either cFH or both cFH and uromodulin proteins. After incubation, the samples were heated with a loading buffer containing β‐mercaptoethanol for 10 min at 100°C and subsequently loaded onto a 10% SDS‐PAGE gel. C3b and its cleavage products were detected by Western blotting using a polyclonal anti‐C3b antibody (Dako Cytomation, Carpinteria, CA, USA). Quantification was performed by densitometry analysis using Image J software (NIH, Bethesda, MD, USA).

### 2.8 Hemolytic Assay of Sheep Erythrocytes

The hemolytic assay was based on a previous study [19]. Serum samples were obtained from three healthy individuals. The positive control consisted of 100% hemolysis of sheep erythrocytes mixed with 7 μg of mouse anti‐human cFH monoclonal antibody (BioPorto Diagnostics A/S) and normal serum. A total of 30 μL of serum was mixed with 7 μg of cFH antibody and incubated at 4°C for 1 h, after which 15 μg of uromodulin was added to each mixture. Subsequently, 4 μg of commercial cFH was included. Finally, the mixtures were diluted with alternative pathway (AP) buffer (containing 144 mM NaCl, 7 mM MgCl_2_, 20 mM Hepes and 10 mM EGTA) to a total volume of 100 μL [28]. Then, 100 μL of sheep erythrocytes (1 × 10^6^ cells/mL in AP buffer) were added to the final mixtures and incubated at 37°C for 40 min. After incubation, the samples were centrifuged at 5000 rpm for 5 min. A total of 50 μL of the supernatant was transferred to a microplate, and the absorbance at 414 nm (A_414_) was measured. Hemolysis (%) was calculated by dividing the A_414_ of the samples by that of total hemolysis (100 μL of sheep erythrocytes mixed with 100 μL of deionised water).

### 2.9 Statistical Analyses

Statistical analyses were conducted using SPSS (version 22.0, Chicago, IL, USA). Quantitative parameters were presented as mean ± SEM. For normally distributed data, one‐way ANOVA was used for comparisons, and post hoc testing was used to analyse the differences between groups. Data were considered statistically significant when *p* < 0.05.

## 3 Results

### 3.1 Characteristics of the Patient With ADTKD‐UMOD and the Influence of Patient‐Derived Uromodulin on cFH Function

**FIGURE 1:**
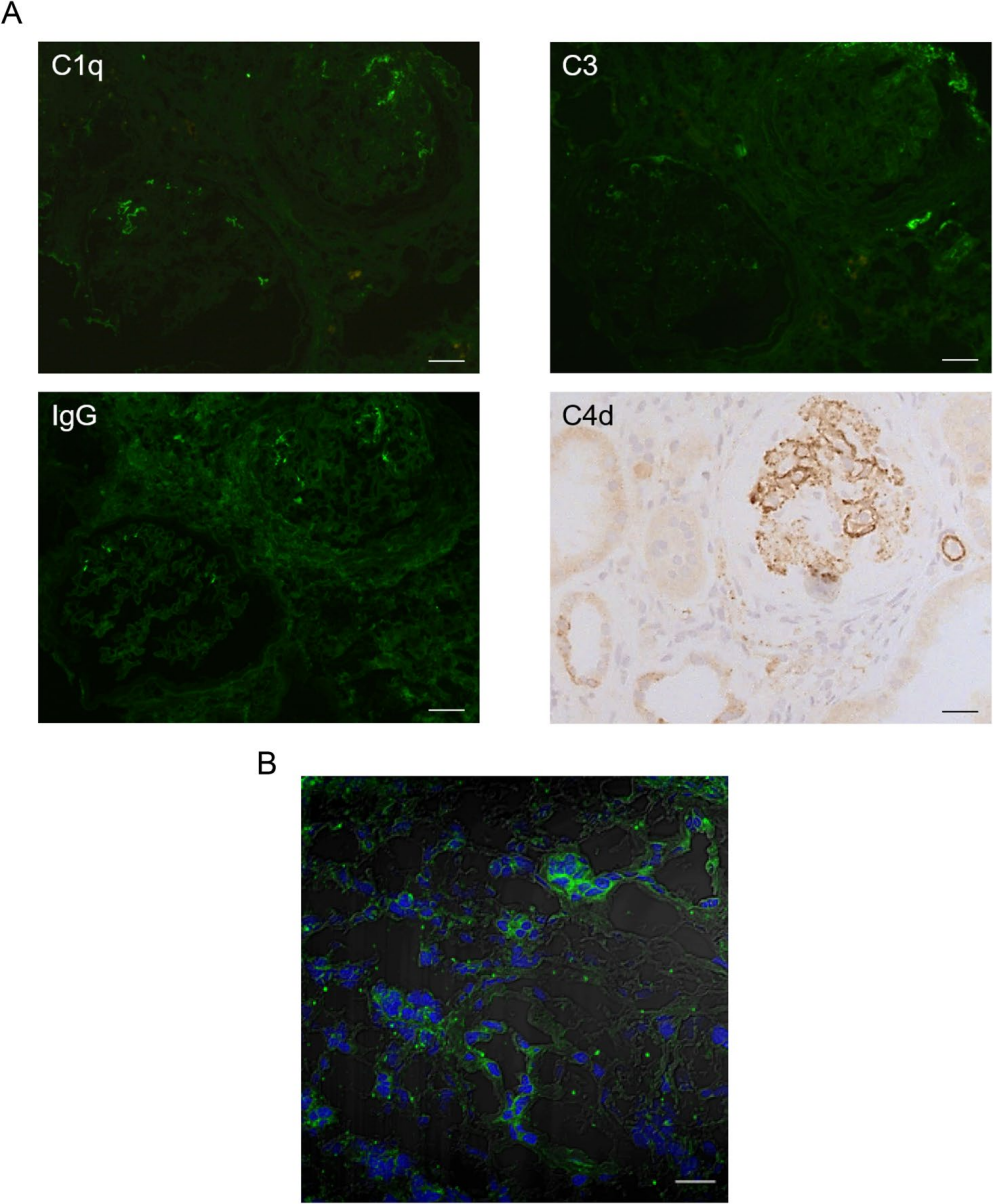
Pathology of the ADTKD patient with complement pathway activation. A renal biopsy and subsequent staining were performed on the patient with ADTKD. (A) Immunofluorescence staining (green) of IgG (+), C3 (+), C1q (+) and immunohistochemical staining for C4d (++) on the renal biopsy tissue from the patient. Scale bar = 40 μm. (B) Immunofluorescence staining (green) of complement factor B (++) on the renal biopsy tissue from the patient. Scale bar = 20 μm.

A 13‐year‐old female patient diagnosed with ADTKD at our hospital was previously reported [29]. Briefly, her laboratory findings included a serum creatinine level of 206.4 μmol/L (eGFR = 26 mL/min/1.73 m^2^) and urinary protein excretion of 0.33 g/day. Serum uric acid was elevated to 385 μmol/L. The patient exhibited a low morning urine osmolarity (312 mOsm/kg). IgG4 levels were slightly elevated to 2.48 g/L, whereas C3 levels decreased to 0.589 g/L. Magnetic resonance imaging of the kidneys revealed the presence of several cysts. Whole‐exome sequencing identified a c.280T>C (p.Cys94Arg) mutation in the *UMOD* gene. The patient denied any relevant family history. Renal biopsy revealed widely distributed glomerulosclerosis, interstitial fibrosis, tubular atrophy and cystic dilation. The interstitium was infiltrated by lymphocytes, monocytes and neutrophils. In contrast to the preimplantation renal biopsy sections from donor kidneys, which were completely negative for complement staining, deposits of IgG (+), C3 (+), C1q (+) and C4d (++) in the patient's kidney were found mainly in the glomerular capillaries and partially within the interstitium (Figure 1A). Notably, no UMOD accumulation was observed in the endoplasmic reticulum [29]. To assess complement activation, factor B staining was conducted, which revealed cFB (++) deposits in tubular epithelial cells and the interstitium (Figure 1B). The patient progressed to end‐stage renal disease (ESRD) within 3 years.

**FIGURE 2:**
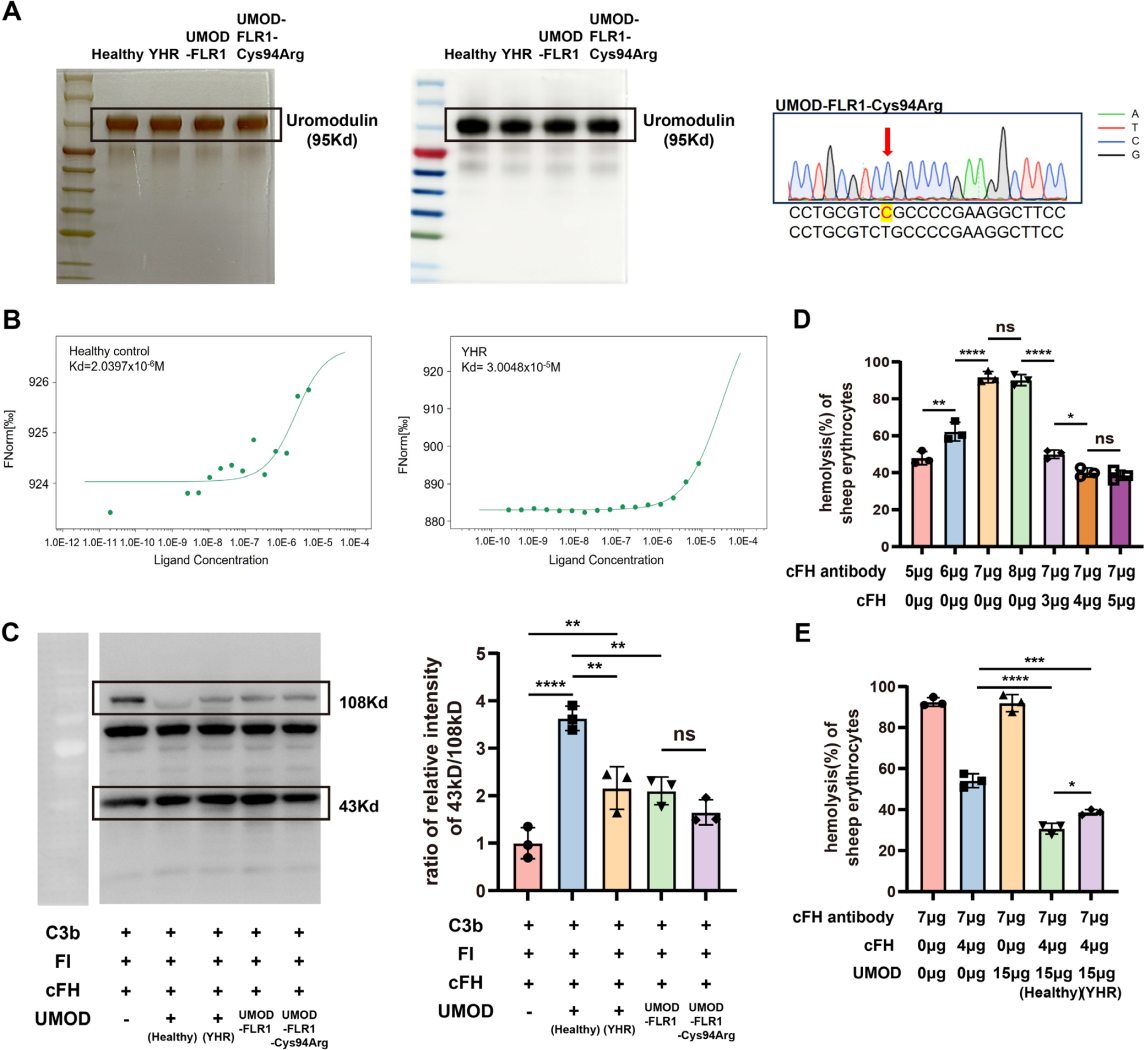
Influence of uromodulin from healthy individuals and the patient with ADTKD on the function of cFH. (A) Purification and identification of uromodulin from healthy individuals and the patient with ADTKD. Western blotting identified the uromodulin proteins; silver staining confirmed the purification of the proteins. Gene sequencing showed that the recombinant uromodulin variant had a c.280T>C (p.C94R) mutation compared to the wild‐type recombinant uromodulin. (B) The binding affinity of uromodulin from healthy individuals and the patient with ADTKD to cFH. Binding was measured using MST. The binding affinities of the uromodulin proteins for cFH are shown in the figure. (C) Western blotting of C3b and its fragments along with densitometric analysis of C3b cleavage. Results are presented as the mean values ± SEM of three independent experiments performed in duplicate wells. The degradation of C3b was calculated as the ratio of the relative intensity of the 43 kDa band to that of the 108 kDa band. *p*‐values are indicated in the figure. (D) Setup of the hemolysis system using sheep erythrocytes. The system included sera from healthy individuals, sheep erythrocytes, and varying levels of cFH antibody and cFH. The experiments were repeated at least 3 times. (E) The inhibitory capacity of uromodulin from a healthy individual and the patient with ADTKD on hemolysis. The experiments were repeated at least 3 times.

We collected and purified uromodulin from the patient's urine (Figure 2A). The binding capacity of patient‐derived uromodulin to cFH (Kd = 3.0048 × 10^−5^ M) was significantly (by five times or more) reduced compared to uromodulin from a healthy control (Kd = 2.0397 × 10^−6^ M) (Figure 2B). Furthermore, the ability of patient‐derived uromodulin to promote the C3b cleavage was reduced compared to that in healthy individuals (Figure 2C). A sheep erythrocyte hemolytic assay was performed to further evaluate cFH function [19]. In this assay, the addition of 7 μg of cFH antibody to 30 μL of healthy human serum resulted in approximately 100% hemolysis, while the addition of 4 μg of exogenous cFH reduced the hemolysis rate to 40%–50% (Figure 2D). Additionally, the introduction of uromodulin from healthy individuals further decreased the hemolysis rate to 30%. Patient‐derived uromodulin did not achieve an inhibition rate similar to that of the healthy controls (Figure 2E).

### 3.2 ADTKD Cohort and Acquisition of Recombinant Uromodulin Proteins

Another five *UMOD* gene mutations used in this study were identified in an ADTKD‐UMOD cohort reported by us previously [24]. This cohort comprised two males and three females, aged between 20 and 44 years. At diagnosis, their serum creatinine levels ranged from 155 to 230 μmol/L, with two patients progressing to ESRD within 2 years after diagnosis (Table 1).

**FIGURE 3:**
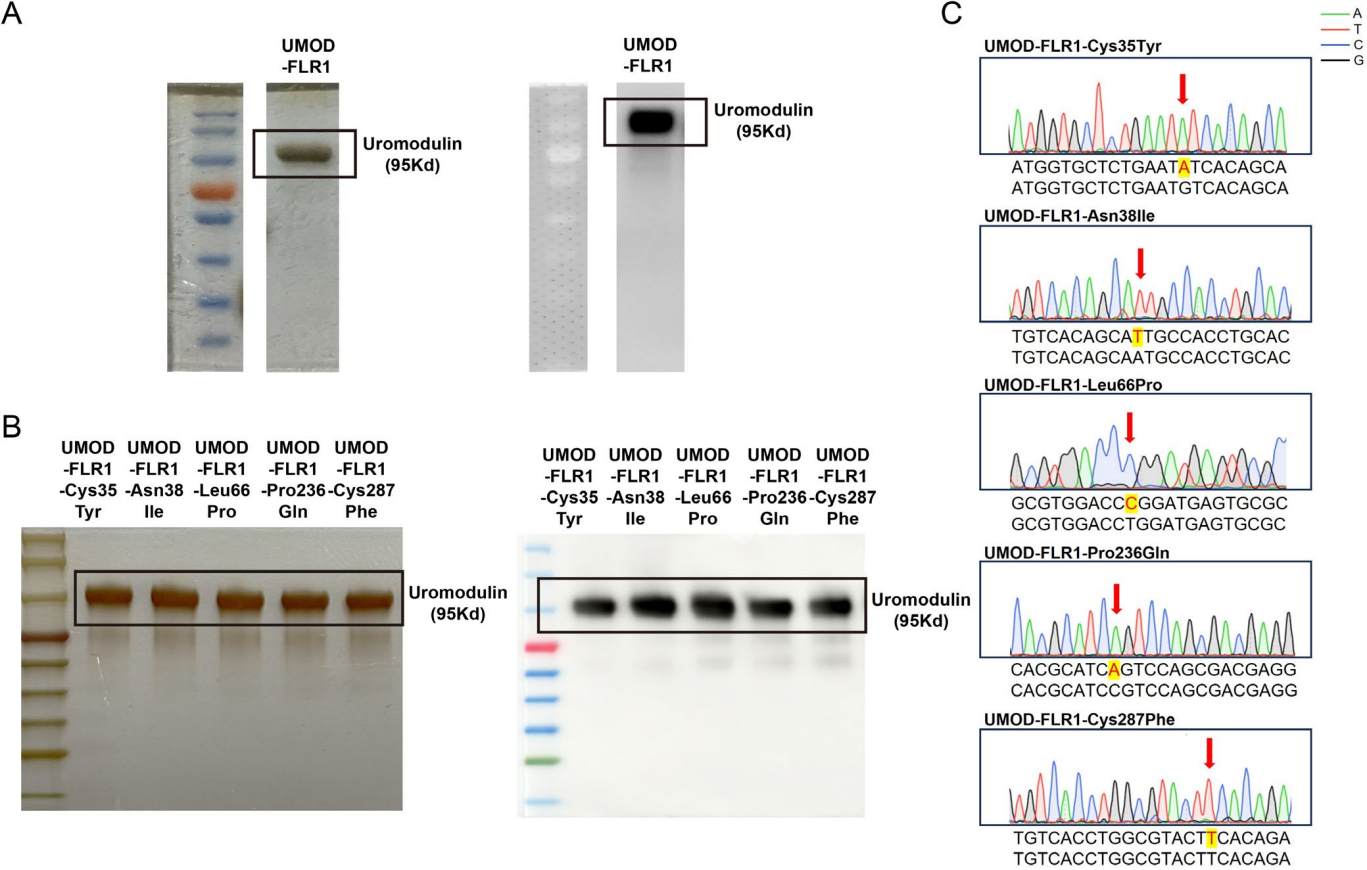
Acquisition of recombinant wild‐type and mutant uromodulin proteins. (A) Purification and identification of recombinant wild‐type uromodulin. Western blotting was used to identify the recombinant wild‐type uromodulin, and silver staining confirmed the purification of recombinant wild‐type uromodulin. (B) Purification and identification of recombinant uromodulin mutants. Western blotting was used to identify the recombinant uromodulin mutants, and silver staining confirmed the purification of recombinant uromodulin mutants. (C) Identification of the mutation sites of recombinant uromodulin mutants. Gene sequencing showed the mutation sites of the recombinant uromodulin mutants compared with the recombinant wild‐type uromodulin, with specific mutation sites indicated in the figure.

To investigate whether other *UMOD* gene mutations also affect the interaction between UMOD and cFH, we constructed and obtained recombinant UMOD proteins: UMOD‐FLR1 and five mutant variants (Cys35Tyr, Asn38Ile, Leu66Pro, Pro236Gln and Cys287Phe). All UMOD proteins were used in subsequent experiments (Figure S1, Figure 3A–C).

### 3.3 Binding Capacity of UMOD‐FLR1 and Its Mutant Variants to cFH

**FIGURE 4:**
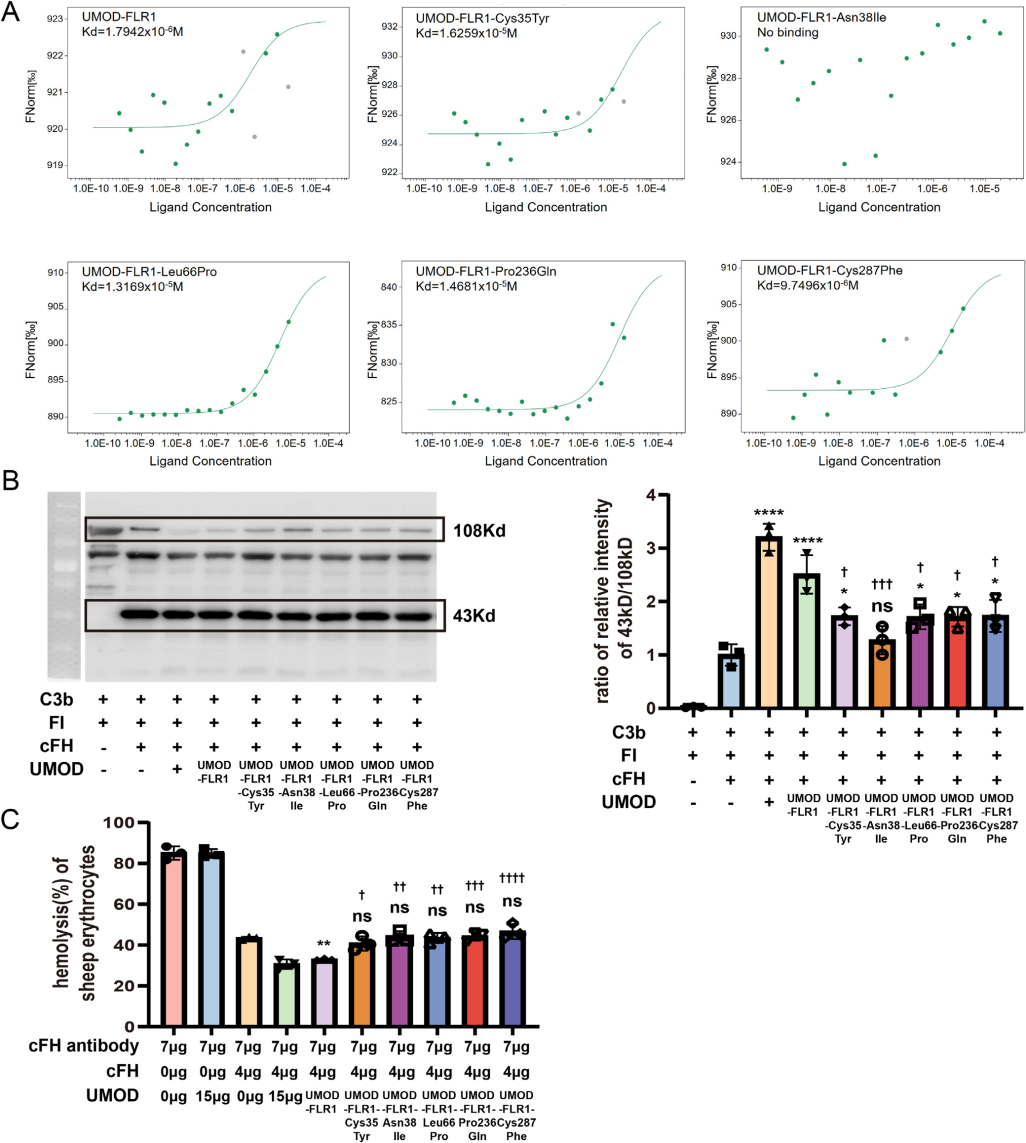
Influence of recombinant wild‐type and mutant uromodulin proteins on the function of cFH. (A) Binding affinity of recombinant uromodulin proteins for cFH. Binding was measured using MST, and the affinities are shown in the figure. (B) Western blotting of C3b and its fragments along with densitometric analysis of C3b cleavage. Lane 1 served as a negative control without cFH. Lane 2 showed the control without UMOD. Lane 3 showed the control with cFH and native UMOD. Lanes 4–8 included different uromodulin proteins, each containing equal amounts of the substance. Results are presented as the mean values ± SEM of three independent experiments performed in duplicate wells. The degradation of C3b was calculated as the ratio of the relative intensity of the 43 kDa band to that of the 108 kDa band. *p*‐values are indicated in the figure (* denotes the significance of each group compared to the control without UMOD, and † denotes the significance of each group compared to the group with UMOD‐FLR1). (C) The inhibitory capacity of natural and recombinant uromodulin proteins on hemolysis. The experiments were repeated at least three times (* denotes the significance of each group compared to the control without UMOD, and † denotes the significance of each group compared to the group with UMOD‐FL).

The dissociation constant (Kd) of UMOD‐FLR1 for cFH was measured to be 1.7942 × 10^−6^ M, which is comparable to that of native uromodulin [20]. Among all mutants, the UMOD‐FLR1‐Asn38Ile variant exhibited no binding to complement factor H. In contrast to the wild‐type UMOD‐FLR1, although the other mutants bound cFH, their binding affinities were reduced, measuring 1.6259 × 10^−5^ M (Cys35Tyr), 1.3169 × 10^−5^ M (Leu66Pro), 1.4681 × 10^−5^ M (Pro236Gln) and 9.7496 × 10^−6^ M (Cys287Phe), respectively (Figure 4A).

The cleavage of C3b was assessed as described previously. Native UMOD and various recombinant UMOD proteins, including UMOD‐FLR1 and its five mutant variants, were evaluated. The ability of UMOD‐FLR1 to promote C3b degradation was slightly lower than that of the native UMOD. Compared with the negative control, the addition of mutants (Cys35Tyr, Leu66Pro, Pro236Gln and Cys287Phe) enhanced the ability of cFH to assist factor I in cleaving C3b, with the exception of Asn38Ile (*p* = 0.8812). However, this effect was significantly reduced compared to that of UMOD‐FLR1 (Figure 4B). Considering that both wild‐type and mutant uromodulin are expected to be present in a patient urine, we also assessed the ability to promote C3b cleavage using a 1:1 mixture of the recombinant wild‐type and mutant proteins in the aforementioned assay system. Compared to wild‐type protein alone, most mutant mixtures exhibited a significantly reduced capacity. The exception was the Cys94Arg mixture, which showed activity comparable to the wild‐type control (Figure S2). Relative to the negative control, UMOD‐FLR1 significantly reduced the hemolysis rate, whereas all mutant UMOD‐FLR1 variants lost their function (Figure 4C).

We also obtained the recombinant uromodulin variant corresponding to patient YHR (Cys94Arg) (Figure 2A), in whose kidney complement factor B deposition was observed. Unlike the natural patient‐derived uromodulin, the recombinant variant was able to assist in C3b cleavage, showing no significant difference from wild‐type UMOD‐FLR1 (Figure 2C).

## 4 Discussion

Tubular atrophy and interstitial fibrosis are well‐established pathological changes associated with ADTKD‐UMOD [30]. Although endoplasmic reticulum stress, cell damage and apoptosis induced by the accumulation of mutant uromodulin have been identified as pathogenic mechanisms in ADTKD‐UMOD [4, 5, 6, 7, 8], additional findings suggest that other mechanisms may also contribute to renal fibrosis [10]. In a patient with genetically confirmed ADTKD‐UMOD, no accumulation of uromodulin in the endoplasmic reticulum was observed [29], indicating that factors other than endoplasmic reticulum stress may be involved in the pathogenic process. We observed deposition of complement C3 and complement factor B in renal tissue, suggesting activation of the complement alternative pathway in ADTKD‐UMOD.

Previous studies have established that complement activation is associated with tubulointerstitial injury [21]. cFH, a critical inhibitory regulator of complement activation, has also been implicated in tubulointerstitial injury and fibrosis by binding to renal tubular epithelial cells and inhibiting complement hyperactivation [23]. In our previous studies, we confirmed that uromodulin can bind to cFH, enhancing its activity as a cofactor for factor I and promoting complement degradation [19, 20]. Therefore, a key question is whether the patient's mutant uromodulin reduces its ability to promote cFH function and whether this decrease is related to local complement activation. To address this, we extracted and purified uromodulin from the patient's urine and confirmed that the mutated uromodulin exhibited decreased binding affinity for cFH and reduced ability to promote cFH to inhibit complement activation.

An interesting question is whether this phenomenon is isolated or common, which remains unclear. In previously reported cases, most patients did not undergo renal biopsy, and urine was not collected. To test this hypothesis, we constructed and expressed a recombinant secreted uromodulin, UMOD‐FLR1, incorporating previously described *UMOD* gene mutations to obtain the mutant UMOD‐FLR1. Our previous study identified five novel mutations in the *UMOD* gene, including p.Cys35Tyr, p.Asn38Ile, p.Leu66Pro, p.Pro236Gln and p.Cys287Phe [24]. Among these mutations, UMOD‐FLR1‐Asn38Ile lost its ability to bind cFH, as well as its capacity to promote complement degradation and inhibit hemolysis in in vitro experiments. The Asn38Ile mutation is located in the EGF‐like domain and directly alters a potential glycosylation site of uromodulin [31]. Given that the deglycosylation of uromodulin reduces its binding to cFH [19], this may explain why UMOD‐FLR1‐Asn38Ile completely lost its affinity for cFH. The other four mutations—Pro236Gln and Cys287Phe, located in the D8C domain, and Cys35Tyr and Leu66Pro, located in the EGF‐like domain—exhibited similar binding affinities. Interestingly, in our experimental system, the difference of the function in promoting complement degradation between UMOD‐FLR1‐Cys94Arg and UMOD‐FLR1 did not reach statistical significance. This finding seems inconsistent with the clinical fact that it is a pathogenic mutation in patients. It's reported that the Cys94 residue is crucial for uromodulin to form dimers and oligomers via disulfide bonds [32, 33, 34].

Natural uromodulin is prone to polymerisation, forming large and heterogeneous oligomers. In the physiological environment of the kidney and urine, uromodulin predominantly exists as a large polymer [35]. According to previous studies, pathogenic mutant isoforms are often retained within cells and impair the polymerisation of the wild‐type protein through a dominant‐negative effect [36]. These evidences strongly suggest that the functional, cFH‐binding form in vivo is likely the correctly assembled polymer. This also explains why the recombinant wild‐type uromodulin showed a significantly reduced ability to promote C3b cleavage compared to native uromodulin purified from healthy human urine in our study. In this work, we aimed to investigate whether mutations impair uromodulin's normal function of regulating complement activation. To achieve this and avoid the complexity introduced by polymerisation, we designed a recombinant monomeric form (UMOD‐FLR1) and its variants that were incapable of polymerisation.

For uromodulin, polymerisation occurs when an external hydrophobic patch (EHP) is cleaved by enzymes, allowing the remaining internal hydrophobic patch (IHP) to facilitate oligomer formation [37]. In theory, a recombinant protein lacking EHP would more closely resemble natural uromodulin. However, this form remains a possibility of polymerisation. Recombinant uromodulin containing EHP (UMOD‐FLR1) does not polymerise into oligomers so that we chose the UMOD‐FLR1 monomer as our research model. As we mentioned above, the Cys94 residue is crucial for uromodulin to form dimers and oligomers. Therefore, we speculate that an important pathogenic mechanism of the natural C94R mutation in patients lies in its impairment of the proper oligomerisation of uromodulin, leading to its aberrant structure. This affects its binding capacity to cFH and its ability to assist C3b cleavage, ultimately resulting in complement overactivation. In contrast, mutations at other sites may directly alter the conformation or key functional surfaces of the uromodulin monomer, thereby affecting its interaction with cFH, which can be detected in our system.

In this study, we used uromodulin directly isolated from patient urine for functional analysis. Compared with an equivalent amount of uromodulin from healthy donors, the patient‐derived protein exhibited a markedly attenuated ability to promote C3b cleavage. The protein sample directly isolated from patient urine was expected to comprise both wild‐type and mutant forms, and although their exact ratio could not be precisely determined, the sample reflected the native composition in the patient. Based on this, we believe that the experimental results can, to some extent, reflect the in vivo situation in patients with ADTKD‐UMOD, where UMOD mutations may lead to impaired C3b cleavage and complement overactivation.

To simulate the heterozygous state in patient urine, we mixed recombinant wild‐type and mutant uromodulin for a functional assay. Since it is challenging to accurately estimate the ratio of the two forms in patient urine, we mixed them at physiologically relevant ratios (1:1) and the ability to promote cFH to assist factor I‐mediated C3b cleavage was still impaired. This suggests that the reduced complement inhibition capacity of the mutant protein itself leads to an overall functional decline in the mixture, even when wild‐type protein is present.

This study has some limitations. Compared to uromodulin derived from healthy individuals, uromodulin extracted from patient urine demonstrated a significantly reduced ability to assist cFH in promoting C3b cleavage and inhibiting hemolysis. However, the performance of the recombinant mutant uromodulin (Cys94Arg) was not significantly different from that of recombinant wild‐type uromodulin. Additionally, although recombinant uromodulin mutants (Cys35Tyr, Asn38Ile, Leu66Pro, Pro236Gln and Cys287Phe) showed a marked reduction in their ability to assist cFH in C3b cleavage compared to UMOD‐FLR1, they still partially assisted cFH function relative to the negative control lacking uromodulin. Nevertheless, their ability to inhibit hemolysis was not significantly different from that of the negative control. These inconsistencies may be due to the non‐polymerising monomeric experimental system employed in this study. Both the recombinant wild‐type and mutant uromodulin proteins exist in monomeric forms, resulting in structural differences from natural uromodulin. Furthermore, natural uromodulin is expressed in the thick ascending limb of Henle's loop [38], whereas recombinant uromodulin proteins are expressed in cell lines, which may also contribute to the differences between natural and recombinant uromodulin. This system is insufficient to fully reveal the potential differences in the C3b cleavage‐promoting functions. Future studies conducted in systems capable of replicating natural oligomerisation will be necessary to validate the impact of the mutations on the structure and function of uromodulin polymers. Furthermore, since ER retention of mutant uromodulin affects its secretion, elucidating the secretion efficiency of each mutant is of great importance. In future studies, we will use dynamic imaging techniques to measure the extent of ER retention of different mutants, which will be an important logical extension of this study.

In summary, the functional studies of natural and recombinant wild‐type and mutant uromodulin suggest that *UMOD* gene mutations may affect the combination of uromodulin and cFH. This could affect the ability of uromodulin to enhance the role of cFH as a cofactor in promoting C3b cleavage, potentially leading to insufficient inhibition of complement system activation, which may be one of the pathogenic mechanisms of ADTKD.

## Author Contributions

Qiuyu Xie: data curation (equal), validation (equal), visualisation (lead), writing – original draft (lead), methodology (equal). Lufeng Bai: data curation (equal), validation (equal), methodology (equal). Kunjing Gong: data curation (equal), validation (equal), methodology (equal). Nan Hu: funding acquisition (equal), investigation (equal). Yuqing Chen: conceptualisation (lead), funding acquisition (equal), investigation (equal), writing – review and editing (lead).

## Funding

This work was supported by the National Key R&D Program of China (No. 2024YFC3406700 and 2024YFC3406705).

## Ethics Statement

This study protocol was reviewed and approved by the Biomedical Research Ethics Committee of Peking University First Hospital (Approval No. 2018‐099). In accordance with national regulations and institutional policies, written informed consent from patients/participants or their legal guardians was not required for participation in this study. For the publication of this case report and any accompanying images, written informed consent was obtained from the patient's parents.

## Consent

Written informed consent was obtained from the parents.

## Conflicts of Interest

The authors declare no conflicts of interest.

## Supporting information


**FIGURE S1:** Design scheme of pCAG‐SP‐6xhis uromodulin expression vector and recombinant uromodulin fragments.
**FIGURE S2:** Effect of mixed recombinant wild‐type and mutant uromodulin proteins on C3b cleavage promoted by factor I and cFH.

## Data Availability

The data that support the findings of this study are available from the corresponding author upon reasonable request.

## References

[1] A. J. Bleyer , K. O. Kidd , M. Živná , and S. Kmoch , “Autosomal Dominant Tubulointerstitial Kidney Disease: A Review,” American Journal of Kidney Diseases 86 (2025): 677–689, 10.1053/j.ajkd.2025.05.015. 40835155

[2] L. Rampoldi , F. Scolari , A. Amoroso , G. Ghiggeri , and O. Devuyst , “The Rediscovery of Uromodulin (Tamm‐Horsfall Protein): From Tubulointerstitial Nephropathy to Chronic Kidney Disease,” Kidney International 80, no. 4 (2011): 338–347, 10.1038/ki.2011.134. 21654721

[3] K. U. Eckardt , S. L. Alper , C. Antignac , et al., “Autosomal Dominant Tubulointerstitial Kidney Disease: Diagnosis, Classification, and Management—A KDIGO Consensus Report,” Kidney International 88, no. 4 (2015): 676–683, 10.1038/ki.2015.28. 25738250

[4] J. Adam , G. Bollée , S. Fougeray , et al., “Endoplasmic Reticulum Stress in UMOD‐Related Kidney Disease: A Human Pathologic Study,” American Journal of Kidney Diseases 59, no. 1 (2012): 117–121, 10.1053/j.ajkd.2011.08.014. 21978600

[5] I. Bernascone , S. Janas , M. Ikehata , et al., “A Transgenic Mouse Model for Uromodulin‐Associated Kidney Diseases Shows Specific Tubulo‐Interstitial Damage, Urinary Concentrating Defect and Renal Failure,” Human Molecular Genetics 19, no. 15 (2010): 2998–3010, 10.1093/hmg/ddq205. 20472742

[6] I. Bernascone , S. Vavassori , A. Di Pentima , et al., “Defective Intracellular Trafficking of Uromodulin Mutant Isoforms,” Traffic 7, no. 11 (2006): 1567–1579, 10.1111/j.1600-0854.2006.00481.x. 17010121

[7] S. W. Choi , O. H. Ryu , S. J. Choi , I. S. Song , A. J. Bleyer , and T. C. Hart , “Mutant Tamm‐Horsfall Glycoprotein Accumulation in Endoplasmic Reticulum Induces Apoptosis Reversed by Colchicine and Sodium 4‐Phenylbutyrate,” Journal of the American Society of Nephrology 16, no. 10 (2005): 3006–3014, 10.1681/asn.2005050461. 16135773

[8] K. Dahan , O. Devuyst , M. Smaers , et al., “A Cluster of Mutations in the UMOD Gene Causes Familial Juvenile Hyperuricemic Nephropathy With Abnormal Expression of Uromodulin,” Journal of the American Society of Nephrology 14, no. 11 (2003): 2883–2893, 10.1097/01.asn.0000092147.83480.b5. 14569098

[9] C. Schaeffer , S. Merella , E. Pasqualetto , D. Lazarevic , and L. Rampoldi , “Mutant Uromodulin Expression Leads to Altered Homeostasis of the Endoplasmic Reticulum and Activates the Unfolded Protein Response,” PLoS One 12, no. 4 (2017): e0175970, 10.1371/journal.pone.0175970. 28437467 PMC5402980

[10] S. E. Williams , A. A. Reed , J. Galvanovskis , et al., “Uromodulin Mutations Causing Familial Juvenile Hyperuricaemic Nephropathy Lead to Protein Maturation Defects and Retention in the Endoplasmic Reticulum,” Human Molecular Genetics 18, no. 16 (2009): 2963–2974, 10.1093/hmg/ddp235. 19465746 PMC2714724

[11] C. Hession , J. M. Decker , A. P. Sherblom , et al., “Uromodulin (Tamm‐Horsfall Glycoprotein): A Renal Ligand for Lymphokines,” Science 237, no. 4821 (1987): 1479–1484, 10.1126/science.3498215. 3498215

[12] D. C. Rhodes , “Binding of Tamm‐Horsfall Protein to Complement 1q Measured by ELISA and Resonant Mirror Biosensor Techniques Under Various Ionic‐Strength Conditions,” Immunology and Cell Biology 78, no. 5 (2000): 474–482, 10.1111/j.1440-1711.2000.t01-3-.x. 11050529

[13] D. C. Rhodes , “Binding of Tamm‐Horsfall Protein to Complement 1q and Complement 1, Including Influence of Hydrogen‐Ion Concentration,” Immunology and Cell Biology 80, no. 6 (2002): 558–566, 10.1046/j.1440-1711.2002.01125.x. 12406390

[14] A. P. Sherblom , N. Sathyamoorthy , J. M. Decker , and A. V. Muchmore , “IL‐2, a Lectin With Specificity for High Mannose Glycopeptides,” Journal of Immunology 143, no. 3 (1989): 939–944. 2787353

[15] C. L. Yu , C. Y. Tsai , W. M. Lin , et al., “Tamm‐Horsfall Urinary Glycoprotein Enhances Monokine Release and Augments Lymphocyte Proliferation,” Immunopharmacology 26, no. 3 (1993): 249–258, 10.1016/0162-3109(93)90041-n. 8288446

[16] D. Cavallone , N. Malagolini , and F. Serafini‐Cessi , “Binding of Human Neutrophils to Cell‐Surface Anchored Tamm‐Horsfall Glycoprotein in Tubulointerstitial Nephritis,” Kidney International 55, no. 5 (1999): 1787–1799, 10.1046/j.1523-1755.1999.00439.x. 10231441

[17] M. D. Säemann , T. Weichhart , M. Zeyda , et al., “Tamm‐Horsfall Glycoprotein Links Innate Immune Cell Activation With Adaptive Immunity via a Toll‐Like Receptor‐4‐Dependent Mechanism,” Journal of Clinical Investigation 115, no. 2 (2005): 468–475, 10.1172/jci22720. 15650774 PMC544039

[18] S. J. Su , K. L. Chang , T. M. Lin , Y. H. Huang , and T. M. Yeh , “Uromodulin and Tamm‐Horsfall Protein Induce Human Monocytes to Secrete TNF and Express Tissue Factor,” Journal of Immunology 158, no. 7 (1997): 3449–3456. 9120306

[19] L. Bai , Q. Xie , M. Xia , et al., “The Importance of Sialic Acid, pH and Ion Concentration on the Interaction of Uromodulin and Complement Factor H,” Journal of Cellular and Molecular Medicine 25, no. 9 (2021): 4316–4325, 10.1111/jcmm.16492. 33788378 PMC8093974

[20] M. Liu , Y. Wang , F. Wang , et al., “Interaction of Uromodulin and Complement Factor H Enhances C3b Inactivation,” Journal of Cellular and Molecular Medicine 20, no. 10 (2016): 1821–1828, 10.1111/jcmm.12872. 27113631 PMC5020621

[21] S. I. Hsu and W. G. Couser , “Chronic Progression of Tubulointerstitial Damage in Proteinuric Renal Disease Is Mediated by Complement Activation: A Therapeutic Role for Complement Inhibitors?,” Journal of the American Society of Nephrology 14, no. Suppl 2 (2003): S186–S191, 10.1097/01.asn.0000070032.58017.20. 12819326

[22] S. de Rodríguez Córdoba , J. Esparza‐Gordillo , E. de Goicoechea Jorge , M. Lopez‐Trascasa , and P. Sánchez‐Corral , “The Human Complement Factor H: Functional Roles, Genetic Variations and Disease Associations,” Molecular Immunology 41, no. 4 (2004): 355–367, 10.1016/j.molimm.2004.02.005. 15163532

[23] B. Renner , V. P. Ferreira , C. Cortes , et al., “Binding of Factor H to Tubular Epithelial Cells Limits Interstitial Complement Activation in Ischemic Injury,” Kidney International 80, no. 2 (2011): 165–173, 10.1038/ki.2011.115. 21544060 PMC3133686

[24] K. Gong , M. Xia , Y. Wang , et al., “Autosomal Dominant Tubulointerstitial Kidney Disease Genotype and Phenotype Correlation in a Chinese Cohort,” Scientific Reports 11, no. 1 (2021): 3615, 10.1038/s41598-020-79331-w. 33574344 PMC7878898

[25] F. Serafini‐Cessi , G. Bellabarba , N. Malagolini , and F. Dall'Olio , “Rapid Isolation of Tamm‐Horsfall Glycoprotein (Uromodulin) From Human Urine,” Journal of Immunological Methods 120, no. 2 (1989): 185–189, 10.1016/0022-1759(89)90241-x. 2500486

[26] M. Brunati , S. Perucca , L. Han , et al., “The Serine Protease Hepsin Mediates Urinary Secretion and Polymerisation of Zona Pellucida Domain Protein Uromodulin,” eLife 4 (2015): e08887, 10.7554/eLife.08887. 26673890 PMC4755741

[27] K. Gong , M. Xia , Y. Wang , et al., “Importance of Glycosylation in the Interaction of Tamm‐Horsfall Protein With Collectin‐11 and Acute Kidney Injury,” Journal of Cellular and Molecular Medicine 24, no. 6 (2020): 3572–3581, 10.1111/jcmm.15046. 32045104 PMC7131921

[28] S. Heinen , A. Hartmann , N. Lauer , et al., “Factor H‐Related Protein 1 (CFHR‐1) Inhibits Complement C5 Convertase Activity and Terminal Complex Formation,” Blood 114, no. 12 (2009): 2439–2447, 10.1182/blood-2009-02-205641. 19528535

[29] M. S. Li , Y. Li , L. Jiang , et al., “ADTKD‐UMOD in a Girl With a de Novo Mutation: A Case Report,” Frontiers in Medicine 9 (2022): 1077655, 10.3389/fmed.2022.1077655. 36606057 PMC9808042

[30] O. Devuyst , E. Olinger , S. Weber , et al., “Autosomal Dominant Tubulointerstitial Kidney Disease,” Nature Reviews. Disease Primers 5, no. 1 (2019): 60, 10.1038/s41572-019-0109-9. 31488840

[31] J. J. van Rooijen , A. F. Voskamp , J. P. Kamerling , and J. F. Vliegenthart , “Glycosylation Sites and Site‐Specific Glycosylation in Human Tamm‐Horsfall Glycoprotein,” Glycobiology 9, no. 1 (1999): 21–30, 10.1093/glycob/9.1.21. 9884403

[32] H. Kajiura , T. Yoshizawa , Y. Tokumoto , et al., “Structure–Function Studies of Ultrahigh Molecular Weight Isoprenes Provide Key Insights Into Their Biosynthesis,” Communications Biology 4, no. 1 (2021), 10.1038/s42003-021-01739-5. PMC7887238 33594248

[33] S. Loguercio , C. Dian , A. Flagiello , et al., “In HspA From *Helicobacter pylori* Vicinal Disulfide Bridges Are a Key Determinant of Domain B Structure,” FEBS Letters 582, no. 23–24 (2008): 3537–3541, 10.1016/j.febslet.2008.09.025. 18805417

[34] D. Oxley and A. Bacic , “Disulphide Bonding in a Stylar Self‐Incompatibility Ribonuclease of *Nicotiana alata* ,” European Journal of Biochemistry 242, no. 1 (1996): 75–80, 10.1111/j.1432-1033.1996.0075r.x. 8954155

[35] F. Serafini‐Cessi , N. Malagolini , and D. Cavallone , “Tamm‐Horsfall Glycoprotein: Biology and Clinical Relevance,” American Journal of Kidney Diseases 42, no. 4 (2003): 658–676, 10.1016/s0272-6386(03)00829-1. 14520616

[36] C. Schaeffer , A. Cattaneo , M. Trudu , et al., “Urinary Secretion and Extracellular Aggregation of Mutant Uromodulin Isoforms,” Kidney International 81, no. 8 (2012): 769–778, 10.1038/ki.2011.456. 22237754

[37] C. Schaeffer , S. Santambrogio , S. Perucca , G. Casari , and L. Rampoldi , “Analysis of Uromodulin Polymerization Provides New Insights Into the Mechanisms Regulating ZP Domain‐Mediated Protein Assembly,” Molecular Biology of the Cell 20, no. 2 (2009): 589–599, 10.1091/mbc.E08-08-0876. 19005207 PMC2626557

[38] S. Bachmann , R. Metzger , and B. Bunnemann , “Tamm‐Horsfall Protein‐mRNA Synthesis Is Localized to the Thick Ascending Limb of Henle's Loop in Rat Kidney,” Histochemistry 94, no. 5 (1990): 517–523, 10.1007/bf00272616. 2283315

