# Supplementary material for: Mutations in UMOD Contribute to the Pathogenesis of ADTKD‐UMOD by Influencing the Function of Complement Factor H
Source: J Cell Mol Med. 2026 Jan 14;30(2):e71025. doi: 10.1111/jcmm.71025 (PMC12800571; doi:10.1111/jcmm.71025)
Supplement: Supplementary file 1 — FIGURE S1: Design scheme of pCAG‐SP‐6xhis uromodulin expression vector and recombinant uromodulin fragments. FIGURE S2: Effect of mixed recombinant wild‐type and mutant uromodulin proteins on C3b cleavage promoted by factor I and cFH. [file JCMM-30-e71025-s001.pdf]

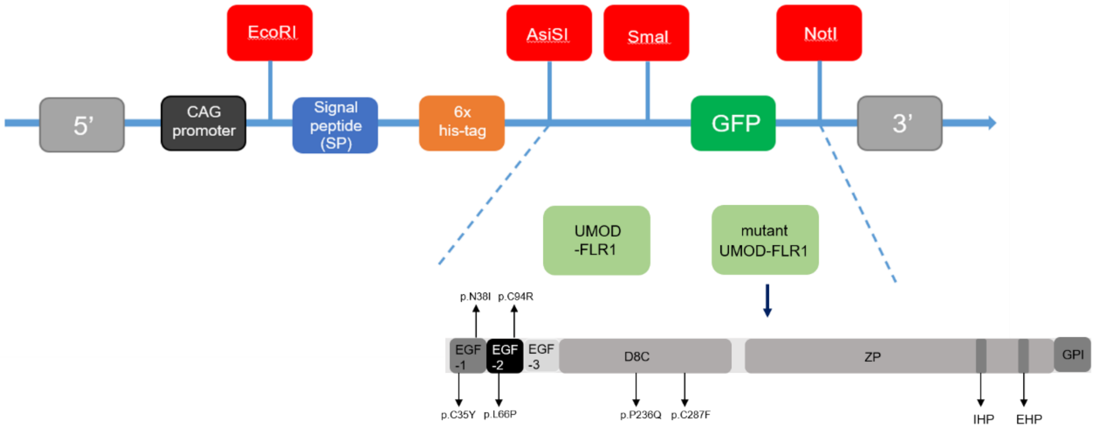

Figure S1. Design scheme of pCAG-SP-6xhis uromodulin expression vector and recombinant uromodulin fragments.

The dark gray square represents the promoter, the blue square represents the signal peptide, the orange square represents the his-tag sequence, the red square represents the enzyme cleavage site, the dark green square represents the replaced GFP fragment, the light green squares represent the recombinant fragments inserted into the vector, and the bottom graphic indicates the mutation sites of uromodulin.

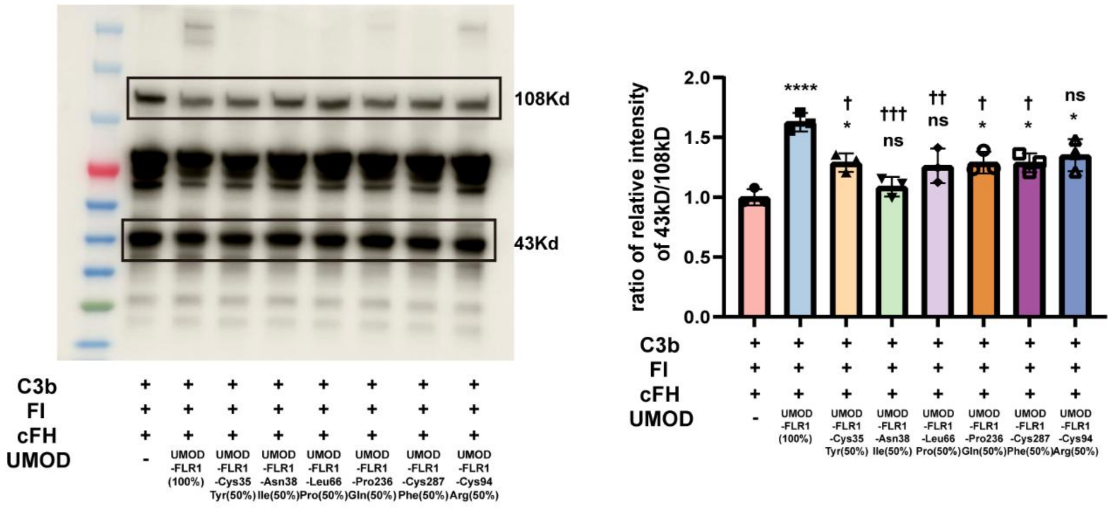

Figure S2. Effect of mixed recombinant wild-type and mutant uromodulin proteins on C3b cleavage promoted by factor I and cFH.

Lane 1 showed the control without UMOD. Lane 2 showed the control with pure UMOD-FLR1. Lanes 3–8 contained the indicated uromodulin variants, each pre-mixed with recombinant wild-type protein at a 1:1 mass ratio. Equal masses of these protein mixtures were loaded in each lane. Results are presented as the mean values ± SEM of 3 independent experiments performed in duplicate wells. The degradation of C3b was calculated as the ratio of the relative intensity of the 43 kDa band to that of the 108 kDa band. P-values are indicated in the figure (* denotes the significance of each group compared to the control without UMOD, and † denotes the significance of each group compared to the group with UMOD-FLR1).

Sequence S1. Recombinant DNA fragments sequences inserted into pCAG-SP-6xHis-GFP vector as substitutes for GFP fragment.

UMOD-FLR1：

GCGATCGC(AsiSI)tcagaagcaagatggtgctctgaatgtcacagcaatgccacctgcacggaggatgaggccgtt
acgacgtgcacctgtcaggagggcttcaccggcgatggcctgacctgcgtggacctggatgagtgcgccattcctggagctcacaac
tgctccgccaacagcagctgcgtaaacacgccaggctccttctcctgcgtctgccccgaaggcttccgcctgtcgcccggtctcggct
gcacagacgtggatgagtgcgctgagcctgggcttagccactgccacgccctggccacatgtgtcaatgtggtgggcagctacttgtg
cgtatgccccgcgggctaccgggggggatggatggcactgtgagtgctccccgggctcctgcgggccggggttggactgcgtgccc
gagggcgacgcgctcgtgtgcgcggatccgtgccaggcgcaccgcaccctggacgagtactggcgcagcaccgagtacggggag
ggctacgcctgcgacacggacctgcgcggctggtaccgcttcgtgggccagggcggtgcgcgcatggccgagacctgcgtgccag
tcctgcgctgcaacacggccgcccccatgtggctcaatggcacgcatccgtccagcgacgagggcatcgtgagccgcaaggcctgc
gcgcactggagcggccactgctgcctgtgggatgcgtccgtccaggtgaaggcctgtgccggcggctactacgtctacaacctgaca
gcgcccccccgagtgtcacctggcgtactgcacagaccccagctccgtggaggggacgtgtgaggagtgcagtatagacgaggactg
caaatcgaataatggcagatggcactgccagtgcaaacaggacttcaacatcactgatatctccctcctggagcacaggctggaatgtg
gggccaatgacatgaaggtgtcgctgggcaagtgccagctgaagagtctgggcttcgacaaggtcttcatgtacctgagtgacagcc
ggtgctcgggcttcaatgacagagacaaccgggactgggtgtctgtagtgaccccagcccgggatggcccctgtgggacagtgttga
cgaggaatgaaacccatgccacttacagcaacaccctctacctggcagatgagatcatcatccgtgacctcaacatcaaaatcaactttg
catgctcctacccccctggacatgaaagtcagcctgaagaccgccctacagccaatggtcagtgctctaaacatcagagtgggcggga
ccggcatgttcaccgtgcggatggcgctcttccagacccccttcctacacgcagccctaccaaggctcctccgtgacactgtccactgag
gcttttctctacgtgggcaccatgttggatgggggcgacctgtcccgatttgcactgctcatgaccaactgctatgccacacccagtagc
aatgccacggacccccctgaagtacttcatcatccaggacagatgcccacacactagagactcaactatccaagtggtggagaatgggg
agtcctcccagggccgattttccgtccagatgttccggtttgctggaaactatgacctagtctacctgcactgtgaagtctatctctgtgac

accatgaatgaaaagtgcaagcctacctgctctgggacc**<u>agattccgaagt</u>**(**CCS**)gggagtgtcatagatcaatcccgt**<u>gtcctg</u>**
<u>aacttgggtcccatcacacggaaa</u>(**EHP**)ggtgtccaggccacagtctga<u>GCGGCCGC(NotI)</u>

SEARWCSECHSNATCTEDEAVTTCTCQEGFTGDGLTCVDLDECAIPGAHNCSAN
SSCVNTPGSFSCVCPEGFRLSPGLGCTDVDECAEPGLSHCHALATCVNVVGSYLCVCP
AGYRGDGWHCECSPGSCGPGLDCVPEGDALVCADPCQAHRTLDEYWRSTEYGEGY
ACDTDLRGWYRFVGQGGARMAETCVPVLRCNTAAPMWLNGTHPSSDEGIVSRKAC
AHWSGHCCLWDASVQVKACAGGYYVYNLTAPPECHLAYCTDPSSVEGTCEECSIDE
DCKSNNGRWHCQCKQDFNITDISLLEHRLECGANDMKVSLGKCQLKSLGFDKVFMY
LSDSRCSGFNDRDNRDWVSVVTPARDGPCGTVLTRNETHATYSNTLYLADEIIIRDLNI
KINFACSYPLDMKVSLKTALQPMVSALNIRVGGTGMFTVRMALFQTPSYTQPYQGSS
VTLSTEAFLYVGTMLDGGDLSRFALLMTNCYATPSSNATDPLKYFIIQDRCPHTRDSTI
QVVENGESSQGRFSVQMFRFAGNYDLVYLHCEVYLCDTMNEKCKPTCSGTRFRSGS
VIDQSRVLNLGPITRKGVQATV

UMOD- Cys35Tyr：

<u>GCGATCGC(AsiSI)</u>tcagaagcaagatggtgctctgaatatcacagcaatgccacctgcacggaggatgaggccgtt
acgacgtgcacctgtcaggagggcttcaccggcgatggcctgacctgcgtggacctggatgagtgcgccattcctggagctcacaac
tgctccgccaacagcagctgcgtaaacacgccaggctccttctcctgcgtctgccccgaaggcttccgcctgtcgcccggtctcggct
gcacagacgtggatgagtgcgctgagcctgggcttagccactgccacgccctggccacatgtgtcaatgtggtgggcagctacttgtg
cgtatgccccgcgggctaccgggggggatggatggcactgtgagtgctccccgggctcctgcgggccggggttggactgcgtgccc
gagggcgacgcgctcgtgtgcgcggatccgtgccaggcgcaccgcaccctggacgagtactggcgcagcaccgagtacggggag
ggctacgcctgcgacacggacctgcgcggctggtaccgcttcgtgggccagggcggtgcgcgcatggccgagacctgcgtgccag

tcctgcgctgcaacacggccgcccccatgtggctcaatggcacgcatccgtccagcgacgagggcatcgtgagccgcaaggcctgc

gcgcactggagcggccactgctgcctgtgggatgcgtccgtccaggtgaaggcctgtgccggcggctactacgtctacaacctgaca

gcgcccccgagtgtcacctggcgtactgcacagaccccagctccgtggaggggacgtgtgaggagtgcagtatagacgaggactg

caaatcgaataatggcagatggcactgccagtgcaaacaggacttcaacatcactgatatctccctcctggagcacaggctggaatgtg

gggccaatgacatgaaggtgtcgctgggcaagtgccagctgaagagtctgggcttcgacaaggtcttcatgtacctgagtgacagcc

ggtgctcgggcttcaatgacagagacaaccgggactgggtgtctgtagtgaccccagcccgggatggcccctgtgggacagtgttga

cgaggaatgaaacccatgccacttacagcaacaccctctacctggcagatgagatcatcatccgtgacctcaacatcaaaatcaactttg

catgctcctacccccctggacatgaaagtcagcctgaagaccgccctacagccaatggtcagtgctctaaacatcagagtgggcggga

ccggcatgttcaccgtgcggatggcgctcttccagaccccttcctacacgcagccctaccaaggctcctccgtgacactgtccactgag

gcttttctctacgtgggcaccatgttggatgggggcgacctgtcccgatttgcactgctcatgaccaactgctatgccacacccagtagc

aatgccacggacccccctgaagtacttcatcatccaggacagatgcccacacactagagactcaactatccaagtggtggagaatgggg

agtcctcccagggccgattttccgtccagatgttccggtttgctggaaactatgacctagtctacctgcactgtgaagtctatctctgtgac

accatgaatgaaaagtgcaagcctacctgctctgggacc**agattccgaagt**(**CCS**)gggagtgtcatagatcaatcccgt**gtcctg**

aacttgggtcccatcacacggaaa(**EHP**)ggtgtccaggccacagtctgaGCGGCCGC(NotI)

SEARWCSEYHSNATCTEDEAVTTCTCQEGFTGDGLTCVDLDECAIPGAHNCSAN

SSCVNTPGSFSCVCPEGFRLSPGLGCTDVDECAEPGLSHCHALATCVNVVGSYLCVCP

AGYRGDGWHCECSPGSCGPGLDCVPEGDALVCADPCQAHRTLDEYWRSTEYGEGY

ACDTDLRGWYRFVGQGGARMAETCVPVLRCNTAAPMWLNGTHPSSDEGIVSRKAC

AHWSGHCCLWDASVQVKACAGGYYVYNLTAPPECHLAYCTDPSSVEGTCEECSIDE

DCKSNNGRWHCQCKQDFNITDISLLEHRLECGANDMKVSLGKCQLKSLGFDKVFMY

LSDSRCSGFNDRDNRDWVSVVTPARDGPCGTVLTRNETHATYSNTLYLADEIIIRDLNI

KINFACSYPLDMKVSLKTALQPMVSALNIRVGGTGMFTVRMALFQTPSYTQPYQGSS

VTLSTEAFLYVGTMLDGGDLSRFALLMTNCYATPSSNATDPLKYFIIQDRCPHTRDSTI

QVVENGESSQGRFSVQMFRFAGNYDLVYLHCEVYLCDTMNEKCKPTCSGTRFRSGS

VIDQSRVLNLGPITRKGVQATV

UMOD- Asn38Ile：

GCGATCGC(AsiSI)tcagaagcaagatggtgctctgaatgtcacagcattgccacctgcacggaggatgaggccgtta

cgacgtgcacctgtcaggagggcttcaccggcgatggcctgacctgcgtggacctggatgagtgcgccattcctggagctcacaact

gctccgccaacagcagctgcgtaaacacgccaggctccttctcctgcgtctgccccgaaggcttccgcctgtcgcccggtctcggctg

cacagacgtggatgagtgcgctgagcctgggcttagccactgccacgccctggccacatgtgtcaatgtggtgggcagctacttgtgc

gtatgccccgcgggctaccgggggggatggatggcactgtgagtgctccccgggctcctgcgggccggggttggactgcgtgcccg

agggcgacgcgctcgtgtgcgcggatccgtgccaggcgcaccgcaccctggacgagtactggcgcagcaccgagtacggggagg

gctacgcctgcgacacggacctgcgcggctggtaccgcttcgtgggccagggcggtgcgcgcatggccgagacctgcgtgccagt

cctgcgctgcaacacggccgcccccatgtggctcaatggcacgcatccgtccagcgacgagggcatcgtgagccgcaaggcctgc

gcgcactggagcggccactgctgcctgtgggatgcgtccgtccaggtgaaggcctgtgccggcggctactacgtctacaacctgaca

gcgccccccgagtgtcacctggcgtactgcacagaccccagctccgtggaggggacgtgtgaggagtgcagtatagacgaggactg

caaatcgaataatggcagatggcactgccagtgcaaacaggacttcaacatcactgatatctccctcctggagcacaggctggaatgtg

gggccaatgacatgaaggtgtcgctgggcaagtgccagctgaagagtctgggcttcgacaaggtcttcatgtacctgagtgacagcc

ggtgctcgggcttcaatgacagagacaaccgggactgggtgtctgtagtgaccccagcccgggatggcccctgtgggacagtgttga

cgaggaatgaaacccatgccacttacagcaacaccctctacctggcagatgagatcatcatccgtgacctcaacatcaaaatcaactttg

catgctcctacccccctggacatgaaagtcagcctgaagaccgccctacagccaatggtcagtgctctaaacatcagagtgggcggga

ccggcatgttcaccgtgcggatggcgctcttccagacccccttcctacacgcagccctaccaaggctcctccgtgacactgtccactgag

gcttttctctacgtgggcaccatgttggatgggggcgacctgtcccgatttgcactgctcatgaccaactgctatgccacacccagtagc

aatgccacggacccccctgaagtacttcatcatccaggacagatgcccacacactagagactcaactatccaagtggtggagaatgggg
agtcctcccagggccgattttccgtccagatgttccggtttgctggaaactatgacctagtctacctgcactgtgaagtctatctctgtgac
accatgaatgaaaagtgcaagcctacctgctctgggacc**agattccgaagt**(**CCS**)gggagtgtcatagatcaatcccgt**gtcctg
aacttgggtcccatcacacggaaa**(**EHP**)ggtgtccaggccacagtctgaGCGGCCGC(NotI)

SEARWCSECHSIATCTEDEAVTTCTCQEGFTGDGLTCVDLDECAIPGAHNCSANS
SCVNTPGSFSCVCPEGFRLSPGLGCTDVDECAEPGLSHCHALATCVNVVGSYLCVCPA
GYRGDGWHCECSPGSCGPGLDCVPEGDALVCADPCQAHRTLDEYWRSTEYGEGYA
CDTDLRGWYRFVGQGGARMAETCVPVLRCNTAAPMWLNGTHPSSDEGIVSRKACA
HWSGHCCLWDASVQVKACAGGYYVYNLTAPPECHLAYCTDPSSVEGTCEECSIDED
CKSNNGRWHCQCKQDFNITDISLLEHRLECGANDMKVSLGKCQLKSLGFDKVFMYL
SDSRCSGFNDRDNRDWVSVVTPARDGPCGTVLTRNETHATYSNTLYLADEIIIRDLNI
KINFACSYPLDMKVSLKTALQPMVSALNIRVGGTGMFTVRMALFQTPSYTQPYQGSS
VTLSTEAFLYVGTMLDGGDLSRFALLMTNCYATPSSNATDPLKYFIIQDRCPHTRDSTI
QVVENGESSQGRFSVQMFRFAGNYDLVYLHCEVYLCDTMNEKCKPTCSGTRFRSGS
VIDQSRVLNLGPITRKGVQATV

UMOD- Leu66Pro：

GCGATCGC(AsiSI)tcagaagcaagatggtgctctgaatgtcacagcaatgccacctgcacggaggatgaggccgtt
acgacgtgcacctgtcaggagggcttcaccggcgatggcctgacctgcgtggacccggatgagtgcgccattcctggagctcacaac
tgctccgccaacagcagctgcgtaaacacgccaggctccttctcctgcgtctgccccgaaggcttccgcctgtcgcccggtctcggct
gcacagacgtggatgagtgcgctgagcctgggcttagccactgccacgccctggccacatgtgtcaatgtggtgggcagctacttgtg
cgtatgccccgcgggctaccgggggggatggatggcactgtgagtgctccccgggctcctgcgggccggggttggactgcgtgccc

gagggcgacgcgctcgtgtgcgcggatccgtgccaggcgcaccgcaccctggacgagtactggcgcagcaccgagtacggggag

ggctacgcctgcgacacggacctgcgcggctggtaccgcttcgtgggccagggcggtgcgcgcatggccgagacctgcgtgccag

tcctgcgctgcaacacggccgcccccatgtggctcaatggcacgcatccgtccagcgacgagggcatcgtgagccgcaaggcctgc

gcgcactggagcggccactgctgcctgtgggatgcgtccgtccaggtgaaggcctgtgccggcggctactacgtctacaacctgaca

gcgcccccgagtgtcacctggcgtactgcacagaccccagctccgtggaggggacgtgtgaggagtgcagtatagacgaggactg

caaatcgaataatggcagatggcactgccagtgcaaacaggacttcaacatcactgatatctccctcctggagcacaggctggaatgtg

gggccaatgacatgaaggtgtcgctgggcaagtgccagctgaagagtctgggcttcgacaaggtcttcatgtacctgagtgacagcc

ggtgctcgggcttcaatgacagagacaaccgggactgggtgtctgtagtgaccccagcccgggatggcccctgtgggacagtgttga

cgaggaatgaaacccatgccacttacagcaacaccctctacctggcagatgagatcatcatccgtgacctcaacatcaaaatcaactttg

catgctcctacccccctggacatgaaagtcagcctgaagaccgccctacagccaatggtcagtgctctaaacatcagagtgggcggga

ccggcatgttcaccgtgcggatggcgctcttccagaccccttcctacacgcagccctaccaaggctcctccgtgacactgtccactgag

gcttttctctacgtgggcaccatgttggatgggggcgacctgtcccgatttgcactgctcatgaccaactgctatgccacacccagtagc

aatgccacggacccccctgaagtacttcatcatccaggacagatgcccacacactagagactcaactatccaagtggtggagaatgggg

agtcctcccagggccgattttccgtccagatgttccggtttgctggaaactatgacctagtctacctgcactgtgaagtctatctctgtgac

accatgaatgaaaagtgcaagcctacctgctctgggacc**<u>agattccgaagt</u>**(**CCS**)gggagtgtcatagatcaatcccgt**<u>gtcctg</u>**

<u>aacttgggtcccatcacacggaaa</u>(**EHP**)ggtgtccaggccacagtctga<u>GCGGCCGC(NotI)</u>

SEARWCSECHSNATCTEDEAVTTCTCQEGFTGDGLTCVDPDECAIPGAHNCSAN

SSCVNTPGSFSCVCPEGFRLSPGLGCTDVDECAEPGLSHCHALATCVNVVGSYLCVCP

AGYRGDGWHCECSPGSCGPGLDCVPEGDALVCADPCQAHRTLDEYWRSTEYGEGY

ACDTDLRGWYRFVGQGGARMAETCVPVLRCNTAAPMWLNGTHPSSDEGIVSRKAC

AHWSGHCCLWDASVQVKACAGGYYVYNLTAPPECHLAYCTDPSSVEGTCEECSIDE

DCKSNNGRWHCQCKQDFNITDISLLEHRLECGANDMKVSLGKCQLKSLGFDKVFMY

LSDSRCSGFNDRDNRDWVSVVTPARDGPCGTVLTRNETHATYSNTLYLADEIIIRDLNI

KINFACSYPLDMKVSLKTALQPMVSALNIRVGGTGMFTVRMALFQTPSYTQPYQGSS

VTLSTEAFLYVGTMLDGGDLSRFALLMTNCYATPSSNATDPLKYFIIQDRCPHTRDSTI

QVVENGESSQGRFSVQMFRFAGNYDLVYLHCEVYLCDTMNEKCKPTCSGTRFRSGS

VIDQSRVLNLGPITRKGVQATV

UMOD- Pro236Gln：

GCGATCGC(AsiSI)tcagaagcaagatggtgctctgaatgtcacagcaatgccacctgcacggaggatgaggccgtt

acgacgtgcacctgtcaggagggcttcaccggcgatggcctgacctgcgtggacctggatgagtgcgccattcctggagctcacaac

tgctccgccaacagcagctgcgtaaacacgccaggctccttctcctgcgtctgccccgaaggcttccgcctgtcgcccggtctcggct

gcacagacgtggatgagtgcgctgagcctgggcttagccactgccacgccctggccacatgtgtcaatgtggtgggcagctacttgtg

cgtatgccccgcgggctaccgggggggatggatggcactgtgagtgctccccgggctcctgcgggccggggttggactgcgtgccc

gagggcgacgcgctcgtgtgcgcggatccgtgccaggcgcaccgcaccctggacgagtactggcgcagcaccgagtacggggag

ggctacgcctgcgacacggacctgcgcggctggtaccgcttcgtgggccagggcggtgcgcgcatggccgagacctgcgtgccag

tcctgcgctgcaacacggccgcccccatgtggctcaatggcacgcatcagtccagcgacgagggcatcgtgagccgcaaggcctgc

gcgcactggagcggccactgctgcctgtgggatgcgtccgtccaggtgaaggcctgtgccggcggctactacgtctacaacctgaca

gcgcccccccgagtgtcacctggcgtactgcacagaccccagctccgtggaggggacgtgtgaggagtgcagtatagacgaggactg

caaatcgaataatggcagatggcactgccagtgcaaacaggacttcaacatcactgatatctccctcctggagcacaggctggaatgtg

gggccaatgacatgaaggtgtcgctgggcaagtgccagctgaagagtctgggcttcgacaaggtcttcatgtacctgagtgacagcc

ggtgctcgggcttcaatgacagagacaaccgggactgggtgtctgtagtgaccccagcccgggatggcccctgtgggacagtgttga

cgaggaatgaaacccatgccacttacagcaacaccctctacctggcagatgagatcatcatccgtgacctcaacatcaaaatcaactttg

catgctcctacccccctggacatgaaagtcagcctgaagaccgccctacagccaatggtcagtgctctaaacatcagagtgggcggga

ccggcatgttcaccgtgcggatggcgctcttccagaccccttcctacacgcagccctaccaaggctcctccgtgacactgtccactgag

gcttttctctacgtgggcaccatgttggatgggggcgacctgtcccgatttgcactgctcatgaccaactgctatgccacacccagtagc

aatgccacggacccccctgaagtacttcatcatccaggacagatgcccacacactagagactcaactatccaagtggtggagaatgggg

agtcctcccagggccgattttccgtccagatgttccggtttgctggaaactatgacctagtctacctgcactgtgaagtctatctctgtgac

accatgaatgaaaagtgcaagcctacctgctctgggacc**agattccgaagt**(**CCS**)gggagtgtcatagatcaatcccgt**gtcctg**

aacttgggtcccatcacacggaaa(**EHP**)ggtgtccaggccacagtctgaGCGGCCGC(NotI)

SEARWCSECHSNATCTEDEAVTTCTCQEGFTGDGLTCVDLDECAIPGAHNCSAN

SSCVNTPGSFSCVCPEGFRLSPGLGCTDVDECAEPGLSHCHALATCVNVVGSYLCVCP

AGYRGDGWHCECSPGSCGPGLDCVPEGDALVCADPCQAHRTLDEYWRSTEYGEGY

ACDTDLRGWYRFVGQGGARMAETCVPVLRCNTAAPMWLNGTHQSSDEGIVSRKAC

AHWSGHCCLWDASVQVKACAGGYYVYNLTAPPECHLAYCTDPSSVEGTCEECSIDE

DCKSNNGRWHCQCKQDFNITDISLLEHRLECGANDMKVSLGKCQLKSLGFDKVFMY

LSDSRCSGFNDRDNRDWVSVVTPARDGPCGTVLTRNETHATYSNTLYLADEIIIRDLNI

KINFACSYPLDMKVSLKTALQPMVSALNIRVGGTGMFTVRMALFQTPSYTQPYQGSS

VTLSTEAFLYVGTMLDGGDLSRFALLMTNCYATPSSNATDPLKYFIIQDRCPHTRDSTI

QVVENGESSQGRFSVQMFRFAGNYDLVYLHCEVYLCDTMNEKCKPTCSGTRFRSGS

VIDQSRVLNLGPITRKGVQATV

UMOD- Cys287Phe：

GCGATCGC(AsiSI)tcagaagcaagatggtgctctgaatgtcacagcaatgccacctgcacggaggatgaggccgtt

acgacgtgcacctgtcaggagggcttcaccggcgatggcctgacctgcgtggacctggatgagtgcgccattcctggagctcacaac

tgctccgccaacagcagctgcgtaaacacgccaggctccttctcctgcgtctgccccgaaggcttccgcctgtcgcccggtctcggct

gcacagacgtggatgagtgcgctgagcctgggcttagccactgccacgccctggccacatgtgtcaatgtggtgggcagctacttgtg

cgtatgccccgcgggctaccggggggatggatggcactgtgagtgctccccgggctcctgcgggccggggttggactgcgtgccc

gagggcgacgcgctcgtgtgcgcggatccgtgccaggcgcaccgcaccctggacgagtactggcgcagcaccgagtacggggag

ggctacgcctgcgacacggacctgcgcggctggtaccgcttcgtgggccagggcggtgcgcgcatggccgagacctgcgtgccag

tcctgcgctgcaacacggccgcccccatgtggctcaatggcacgcatccgtccagcgacgagggcatcgtgagccgcaaggcctgc

gcgcactggagcggccactgctgcctgtgggatgcgtccgtccaggtgaaggcctgtgccggcggctactacgtctacaacctgaca

gcgccccccgagtgtcacctggcgtacttcacagaccccagctccgtggaggggacgtgtgaggagtgcagtatagacgaggactg

caaatcgaataatggcagatggcactgccagtgcaaacaggacttcaacatcactgatatctccctcctggagcacaggctggaatgtg

gggccaatgacatgaaggtgtcgctgggcaagtgccagctgaagagtctgggcttcgacaaggtcttcatgtacctgagtgacagcc

ggtgctcgggcttcaatgacagagacaaccgggactgggtgtctgtagtgaccccagcccgggatggcccctgtgggacagtgttga

cgaggaatgaaacccatgccacttacagcaacaccctctacctggcagatgagatcatcatccgtgacctcaacatcaaaatcaactttg

catgctcctacccccctggacatgaaagtcagcctgaagaccgccctacagccaatggtcagtgctctaaacatcagagtgggcggga

ccggcatgttcaccgtgcggatggcgctcttccagaccccttcctacacgcagccctaccaaggctcctccgtgacactgtccactgag

gcttttctctacgtgggcaccatgttggatgggggcgacctgtcccgatttgcactgctcatgaccaactgctatgccacacccagtagc

aatgccacggacccccctgaagtacttcatcatccaggacagatgcccacacactagagactcaactatccaagtggtggagaatgggg

agtcctcccagggccgattttccgtccagatgttccggtttgctggaaactatgacctagtctacctgcactgtgaagtctatctctgtgac

accatgaatgaaaagtgcaagcctacctgctctgggacc**agattccgaagt**(**CCS**)gggagtgtcatagatcaatcccgt**gtcctg**

aacttgggtcccatcacacggaaa(**EHP**)ggtgtccaggccacagtctgaGCGGCCGC(NotI)

SEARWCSECHSNATCTEDEAVTTCTCQEGFTGDGLTCVDLDECAIPGAHNCSAN

SSCVNTPGSFSCVCPEGFRLSPGLGCTDVDECAEPGLSHCHALATCVNVVGSYLCVCP

AGYRGDGWHCECSPGSCGPGLDCVPEGDALVCADPCQAHRTLDEYWRSTEYGEGY

ACDTDLRGWYRFVGQGGARMAETCVPVLRCNTAAPMWLNGTHPSSDEGIVSRKAC

AHWSGHCCLWDASVQVKACAGGYYVYNLTAPPECHLAYFTDPSSVEGTCEECSIDE

DCKSNNGRWHCQCKQDFNITDISLLEHRLECGANDMKVSLGKCQLKSLGFDKVFMY

LSDSRCSGFNDRDNRDWVSVVTPARDGPCGTVLTRNETHATYSNTLYLADEIIIRDLNI

KINFACSYPLDMKVSLKTALQPMVSALNIRVGGTGMFTVRMALFQTPSYTQPYQGSS

VTLSTEAFLYVGTMLDGGDLSRFALLMTNCYATPSSNATDPLKYFIIQDRCPHTRDSTI

QVVENGESSQGRFSVQMFRFAGNYDLVYLHCEVYLCDTMNEKCKPTCSGTRFRSGS

VIDQSRVLNLGPITRKGVQATV

The bases and amino acids identified in red font on a yellow background represent the mutation sites of the inserted fragments.
